# Perioperative immune-related adverse events in gastric cancer: a new challenge for surgical safety in the era of neoadjuvant immunotherapy

**DOI:** 10.1093/gastro/goag045

**Published:** 2026-05-01

**Authors:** Zhouqiao Wu, Jingpu Wang, Ziyu Li, Jiafu Ji

**Affiliations:** Key Laboratory of Carcinogenesis and Translational Research (Ministry of Education), Department of Gastrointestinal Surgery, Peking University Cancer Hospital and Institute, Beijing, 100142, P. R. China; Department of Thoracic Surgery, The First Affiliated Hospital of Soochow University, Suzhou, Jiangsu, 215006, P. R. China; Institute of Thoracic Surgery, The First Affiliated Hospital of Soochow University, Suzhou, Jiangsu, 215006, P. R. China; State Key Laboratory of Holistic Integrative Management of Gastrointestinal Cancers, Key Laboratory of Carcinogenesis and Translational Research (Ministry of Education), Department of Gastrointestinal Surgery, Peking University Cancer Hospital and Institute, Beijing, 100142, P. R. China; State Key Laboratory of Holistic Integrative Management of Gastrointestinal Cancers, Key Laboratory of Carcinogenesis and Translational Research (Ministry of Education), Department of Gastrointestinal Surgery, Peking University Cancer Hospital and Institute, Beijing, 100142, P. R. China

As surgical oncologists, we have been eagerly awaiting the results of the Matterhorn trial, which have now been officially published [1]. This landmark study is poised to transform global treatment strategies for gastric cancer and substantially improve patient outcomes. At the same time, however, it has also become clear that surgical safety in the era of neoadjuvant chemotherapy and immunotherapy is facing new challenges. In the Matterhorn trial, the perioperative mortality rate approached 5% [1], which is notably higher and somewhat inconsistent with the results of previous reports [2, 3]. Taken together, these perioperative complications suggest that surgical safety in gastric cancer today requires a more multidimensional assessment.

## Perioperative adverse events of immunotherapy

Other modalities of preoperative therapy have also revolutionized gastric cancer treatment. For example, the CROSS regimen has become standard in many Western countries for EGJ tumors, and its effects on local tissue edema and fibrosis are well recognized [4]. While neoadjuvant radiotherapy may increase surgical difficulty, its survival benefits have firmly established it as standard care.

Immunotherapy has already reshaped the treatment landscape for gastric cancer, progressing from later-line therapy in advanced disease to frontline treatment and, more recently, to perioperative use. The Matterhorn trial demonstrated that neoadjuvant chemoimmunotherapy significantly improves event-free survival compared with chemotherapy alone in patients with gastric or gastroesophageal junction adenocarcinoma [1]. However, the integration of immunotherapy also raises new concerns regarding immune-related adverse events (irAEs). Unlike patients with advanced disease, candidates for neoadjuvant therapy are potentially curable with surgery. For these patients, severe irAEs that preclude surgery could be detrimental to their prognosis.

Encouragingly, the Matterhorn trial showed that neoadjuvant chemoimmunotherapy was as tolerable and safe as perioperative fluorouracil plus leucovorin, oxaliplatin, and docetaxel (FLOT) [1]. Yet, the trial only included relatively fit patients (ECOG 0–1, adequate organ and marrow function), with those ≥75 years comprising only 8% of the cohort. In real-world practice, trial findings are often extrapolated to broader populations. An observational study has shown that frail or elderly patients face higher risks of incomplete therapy and loss of the opportunity to undergo surgery [5]. Whether the addition of immunotherapy may further exacerbate this risk remains an open question, underscoring the need for real-world validation. Consequently, careful consideration of patient selection criteria should be incorporated into future guidelines.

In addition, the interplay between irAEs and surgery warrants attention. In the past two years, we have encountered several patients whose previously recovered irAEs, such as checkpoint inhibitor pneumonitis, recurred shortly after surgery or who developed severe de novo irAEs such as adrenal insufficiency in the immediate postoperative period. These events present unique challenges for perioperative management. Compared with the chemotherapy era, perioperative assessment and management in patients receiving immunotherapy must be more comprehensive. Beyond routine hematologic evaluations, preoperative assessments should include cardiac function, chest computed tomography, and hypothalamic–pituitary–adrenal axis hormone levels. Postoperatively, clinicians must remain vigilant for endocrine-related complications, such as adrenal insufficiency, when patients present with nonspecific symptoms like fatigue or hypotension.

In the perioperative management of gastric cancer treated with neoadjuvant immunotherapy, clinicians should be alert not only to conventional postoperative complications but also to irAEs [6, 7]. Endocrine toxicities, particularly hypophysitis, adrenal insufficiency, and thyroid dysfunction, are among the most easily overlooked because they often present with nonspecific findings such as fatigue, hypotension, hyponatremia, and altered mental status, which may mimic postoperative weakness, infection, or volume depletion [6–8]. Checkpoint inhibitor pneumonitis may present with cough, dyspnea, fever, or hypoxemia and should be differentiated from pneumonia, atelectasis, and pulmonary embolism [6, 7]. Immune-mediated hepatitis, pancreatitis, and myocarditis are less common but potentially serious toxicities that may substantially affect perioperative safety if not recognized promptly [6, 7]. In patients who develop persistent fever, refractory hypotension, unexplained hypoxemia, electrolyte disturbances, or unexpected enzyme abnormalities after surgery, irAEs should be considered in parallel with surgical complications [6, 7, 9]. Management should emphasize preoperative baseline screening and symptom-triggered postoperative evaluation [6, 7, 9]. For suspected moderate-to-severe irAEs, immune checkpoint inhibitors should be withheld promptly, multidisciplinary consultation should be initiated early, and corticosteroids or hormone replacement should be administered according to the type of toxicity [6, 7]. Perioperative stress-dose glucocorticoids are particularly important when adrenal insufficiency is present or strongly suspected [6–9].

Overall, while most randomized studies to date suggest that the addition of immunotherapy does not substantially increase perioperative risk compared with chemotherapy, these trials are limited by low event rates and the selective enrollment of relatively healthy patients. Robust real-world studies are essential to delineate the optimal patient population for perioperative chemoimmunotherapy and to refine evidence-based clinical guidelines. In addition, the establishment of more comprehensive preoperative evaluation criteria for patients may further reduce the occurrence of severe irAEs.
